# Noncoding human Y RNAs are overexpressed in tumours and required for cell proliferation

**DOI:** 10.1038/sj.bjc.6604254

**Published:** 2008-02-19

**Authors:** C P Christov, E Trivier, T Krude

**Affiliations:** 1 Department of Zoology, University of Cambridge, Downing Street, Cambridge CB2 3EJ, UK; 2 CRT Development Laboratory, Cancer Research Technology, Wolfson Institute for Biomedical Research, The Cruciform Building, University College London, Gower Street, London WC1E 6BT, UK

**Keywords:** noncoding RNA, DNA replication, carcinoma, cancer biomarker, cell proliferation

## Abstract

Noncoding Y RNAs have recently been identified as essential factors for chromosomal DNA replication in human cell nuclei. Here, we investigate the expression of human Y RNAs in tumours and test their requirement for cell proliferation. Relative expression levels of all four human Y RNAs (hY1, hY3, hY4 and hY5 RNA) were determined by quantitative RT–PCR in extracts from human solid tumours, corresponding nonmalignant normal tissues and derived cultured cells. On average, all four hY RNAs are significantly overexpressed in solid tumours between 4- and 13-fold, compared to the corresponding normal tissues. In particular, hY1 and hY3 RNAs are overexpressed in carcinomas (and adenocarcinomas) of the bladder, cervix, colon, kidney, lung and prostate with extremely high statistical significance (ANOVA, between groups, *P*<10e-22). A functional requirement of all four hY RNAs for cell proliferation was investigated in a systematic survey for loss-of-function by RNA interference (RNAi). Degradation of hY1 and hY3 RNAs in human cell lines resulted in a significant cytostatic inhibition of cell proliferation. We conclude that noncoding hY RNAs have potential both as new cancer biomarkers and as molecular targets for anti-proliferative intervention.

Cancer is a disease characterised by uncontrolled cell proliferation at the wrong time and place in a given tissue. Chromosomal DNA replication is the major driving force for cell proliferation both during normal development and in cancer. Proteins directly involved in chromosome replication provide molecular biomarkers for cell proliferation and cancer. For instance, antibodies specific for Cdc6 and MCM proteins allow the detection of proliferating cells in tumours and normal tissues with high specificity (Todorov *et al*, 1998; Williams *et al*, 1998; Freeman *et al*, 1999). The most widely used proliferation biomarker in clinical histopathology, however, is probably Ki-67. Antibodies specific for Ki-67 protein have been established as useful diagnostic biomarkers for identifying proliferating cells within a given cell population (reviewed by Scholzen and Gerdes, 2000; Brown and Gatter, 2002). The cellular function of Ki-67 protein is still unclear, but depletion of Ki-67 protein by antisense oligonucleotides directed against Ki-67 mRNA leads to an inhibition of cell proliferation and tumour growth, suggesting that Ki-67 protein is functionally involved in cell cycle progression and may be a potential target in anticancer therapy (Kausch *et al*, 2003).

In addition to proteins, noncoding RNAs are also involved in the regulation of most biological processes (reviewed by Michel, 2002; Storz and Wassarman, 2004; Bernstein and Allis, 2005), therefore making them highly relevant for cancer research. It has recently been established that the expression patterns of noncoding microRNAs (miRNAs) are altered in many human cancers (reviewed by Garzon *et al*, 2006; Hammond, 2006; Osada and Takahashi, 2007). Several miRNAs are overexpressed in cancers, such as the *miR-17-92* cluster in lymphomas and cancers of the lung, prostate, colon and breast, suggesting that they may function as oncogenes. Conversely, the expression of some miRNAs is reduced in malignancies, such as *let-7* in lung cancers, suggesting that miRNAs can function as tumour suppressors. Consequently, expression patterns of noncoding miRNAs may be used as proliferation biomarkers, and they could even be used as potential targets for therapeutic intervention. A pioneering study in mice has established that silencing of miRNA levels is feasible *in vivo* by injection of modified antisense oligonucleotides called ‘antagomirs*’* (Krutzfeldt *et al*, 2005).

A different class of noncoding RNAs termed Y RNAs has been shown to be functionally required for chromosomal DNA replication in mammalian cell nuclei (Christov *et al*, 2006). Y RNAs were originally identified as the RNA component of soluble ribonucleoproteins, which are detected by sera from patients suffering from systemic lupus erythematosis (Lerner *et al*, 1981). Four Y RNAs are present in human cells: hY1, hY3, hY4 and hY5, ranging in size from 83 to 112 nucleotides (Hendrick *et al*, 1981), and they fold into characteristic stem-loop structures (O’Brien *et al*, 1993; van Gelder *et al*, 1994). Each hY RNA is encoded by a single functional gene, which is transcribed by RNA polymerase III from an upstream type 3 promoter (Hendrick *et al*, 1981; Maraia *et al*, 1994, 1996; Matera *et al*, 1995). The Y RNA genes have been conserved during vertebrate evolution, even though different numbers of active Y RNA genes exist in different species due to gene losses, duplications and rearrangements (Mosig *et al*, 2007; Perreault *et al*, 2007). Our recent observation that hY RNAs are functionally required for semiconservative mammalian chromosomal DNA replication (Christov *et al*, 2006) warrants an investigation into whether Y RNAs may also play a role in cell proliferation and cancer.

In this study, we analyse the expression of hY RNAs in human tumours and corresponding nonmalignant tissues by quantitative real-time PCR, and investigate their functional requirement for cell proliferation in human cell lines by RNA interference. Our results show that human Y RNAs are significantly overexpressed in human solid tumours, and their degradation results in an inhibition of cell proliferation.

## MATERIALS AND METHODS

### Cell culture

All cell lines were propagated as subconfluent monolayer cultures in DMEM-medium, supplemented with 10% foetal bovine serum, 10 U ml^−1^ penicillin, 0.1 mg ml^−1^ streptomycin and 2.5 *μ*g ml^−1^ amphotericin B (Fungizone) (all Gibco Invitrogen, Carlsbad, CA, USA). Cell viability was assessed by exclusion of trypan blue (Sigma-Aldrich, St Louis, MO, USA), as described (Nabatiyan *et al*, 2006).

### RNA samples

#### Pools of tissue-specific RNA

A panel of total RNA purified from pools of 20 different human normal tissues (FirstChoice® Human Total RNA Survey Panel, NO AM6000) was purchased from Ambion (Ambion-ABI, Austin, TX, USA). Each pool contains purified total RNA from three tissue donors.

#### Individual samples from specific anatomic sites

RNA samples purified from specific anatomic sites of anonymous patients were purchased from Clinomics BioSciences (Watervliet, NY, USA). Each sample is derived from a defined anatomical site of healthy or cancer origin (carcinoma and adenocarcinoma), obtained from surgeries.

#### Soluble RNA from human cultured cells

Total soluble RNA was obtained from human cultured cells by hypotonic extraction and purified by phenol extraction and ethanol precipitation, as described (Christov *et al*, 2006).

### Quantitative RT–PCR

cDNA was synthesised from commercially obtained pure RNA samples using random sequence hexamer primers (Sigma-Genosys, Haverhill, Suffolk, UK), or from RNA purified from human cultured cells using a set of specific primers complementary to the 3′ ends of all tested RNAs as described (Christov *et al*, 2006).

Relative RNA expression levels in extracts from human tissues were determined by quantitative PCR on a PRISM 7900HT sequence detection system (Applied Biosystems Incorporated, ABI, Foster City, CA, USA), with the following settings: 10 min at 96°C, 15 s at 96°C and 1 min at 60°C for 50 cycles. The MKI67 TaqMan Assay (Assay ID Hs00606991_m1; ABI) was used for amplification of Ki67 cDNA, and the HPRTI TaqMan Assay (Assay ID 4326321E; ABI) was used for amplification of HPRT cDNA. The TaqMan Universal PCR Master Mix (ABI) was used for these amplification reactions. The SYBR Green PCR Master Mix (ABI) was used for the hY RNA-derived cDNA templates, using the hY RNA-specific primer pairs (200 nM) described previously (Christov *et al*, 2006).

Relative RNA expression levels in extracts from cultured human cells were determined by quantitative PCR on the iCycler iQ platform (Bio-Rad, Hercules, CA, USA), using the iQ SYBR® Green Supermix labelling kit (BioRad), using conditions and primer pairs exactly as described previously (Christov *et al*, 2006). In addition, Ki67 cDNA was amplified by the following primer pair: Ki67a: CAGGTCAGGAAGGTCTACAG, Ki67b: TTGTTGTAGTAGTGTTGCCT (Sigma-Genosys).

For each sample, the differences in threshold cycles between the experimental RNAs and a calibrator RNA (HPRT mRNA) were determined as follows: ΔC_*t*_=C_*t *experimental_−C_*t* calibrator_.

Mean values of at least three parallel sets of data acquisitions were used per data point. Relative expression levels were determined from the mean ΔC_*t*_ values as follows: relative expression level=2 exp ΔC_*t*_.

### Statistics

Spearman's rank correlations and analysis of variance (ANOVA) of ΔC_*t*_ values were computed using the R software package (http://www.r-project.org). *T*-tests were performed using Microsoft Excel.

### RNA interference *in vivo*

Primer pair sequences used to direct generation of siRNAs *in vitro* are detailed in the supplementary material. Individual siRNAs were chemically synthesised using an Ambion Silencer® siRNA construction kit as detailed previously (Nabatiyan and Krude, 2004). Transfections were performed with 10 nM siRNAs using Lipofectamine™ 2000 reagent (Invitrogen, Carlsbad, CA, USA) and OptiMEM® (Gibco Invitrogen), as specified by the supplier. Identical concentrations of Lipofectamine were used for all siRNAs.

## RESULTS

### Expression profiles of hY RNAs in human tissues

Expression levels for each of the four hY RNAs were determined by quantitative RT–PCR and expression levels were normalised to HPRT mRNA, which shows very low variation in expression levels between different human tissues and cell types (Vandesompele *et al*, 2002; de Kok *et al*, 2005). We initially determined the relative expression levels of all four hY RNAs in a selection of 20 nonmalignant human tissues, and compared them to the levels of the proliferation biomarker Ki-67 mRNA.

**Figure 1:**
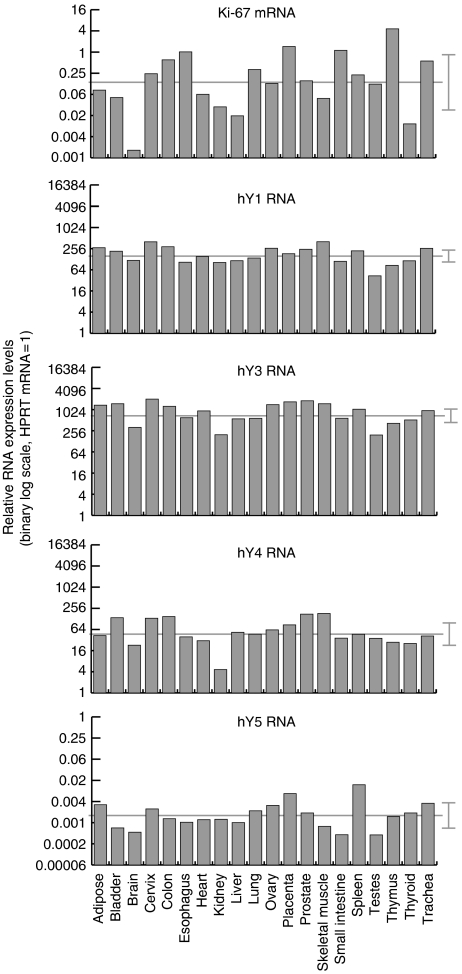
Relative expression levels of hY RNAs in human tissues. Expression levels of the indicated RNAs were determined by qRT–PCR relative to HPRT mRNA in extracts from 20 different human tissues. Mean values of three separate data acquisitions per tissue sample are shown. The expression level of HPRT mRNA is set as one. Mean relative expression values for each RNA across all tissues examined are indicated by a horizontal light grey line, and s.d. from the mean (± std) by brackets to the right of each panel.

All four hY RNAs and Ki-67 mRNA were expressed in all tissues investigated (Figure 1). The relative expression levels of the four individual hY RNAs varied far less between different tissues than Ki-67 mRNA (Figure 1). The three large hY RNAs hY1, hY3 and hY4 showed significantly and positively correlated relative expression levels across different tissue types (Spearman's rank correlation coefficients: hY1 *vs* hY3, Rs=0.833, *P*=2.2 × 10^−16^; hY1 *vs* hY4, Rs=0.747, *P*=2.3 × 10^−4^; hY3 *vs* hY4, Rs=0.839, *P*=2.2 × 10^−16^). hY5 expression levels were positively but nonsignificantly correlated to the other three hY RNAs (hY1 *vs* hY5, Rs=0.472, *P*=0.037; hY3 *vs* hY5, Rs=0.449, *P*=0.048; hY4 *vs* hY5, Rs=0.220, *P*=0.349). Finally, the hY RNA expression levels were either not correlated or were positively but nonsignificantly correlated with Ki67 mRNA expression levels (hY1 *vs* Ki67, Rs=−0.003, *P*=0.992; hY3 *vs* Ki67, Rs=0.239, *P*=0.307; hY4 *vs* Ki67, Rs=0.186, *P*=0.428; hY5 *vs* Ki67, Rs=0.320, *P*=0.167). The observation that some pairs are strongly correlated while others are substantially weaker allows two basic conclusions. First, expression levels of hY RNAs do vary with tissue type. Second, while the expression of hY1, hY3 and hY4 RNAs are to some extent linked, expression of hY5 RNA and Ki67 mRNA appear largely separate.

### Elevated hY RNA expression in human tumours

**Figure 2:**
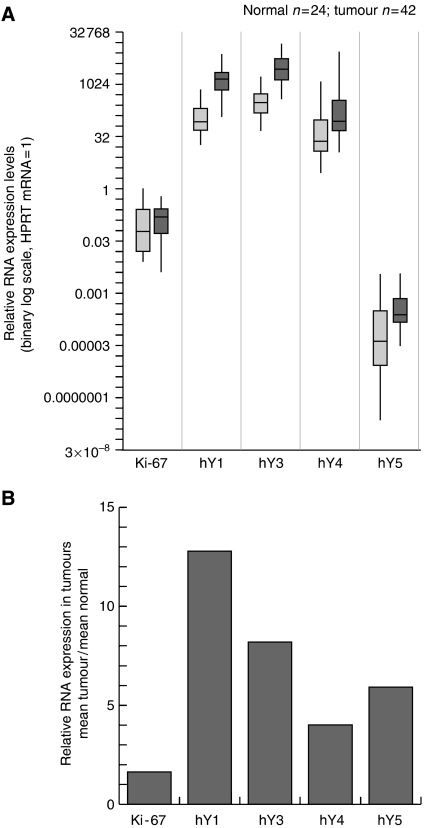
Elevated relative expression of hY RNAs in human cancer tissue compared to corresponding normal nonmalignant tissues. Expression levels of the indicated RNAs were determined by qRT–PCR relative to HPRT mRNA in extracts from 24 individual normal tissue samples and 42 individual carcinomas and adenocarcinomas. (**A**) Distributions of relative RNA expression levels. Box and whisker plots are shown for the distributions of relative expression levels for each RNA as indicated. Thin vertical lines indicate range, boxes indicate the 25th–75th percentile and black horizontal lines indicate the median for each distribution. Light grey boxes represent normal sample distributions, dark grey boxes represent tumour sample distributions. (**B**) Elevated hY RNA expression in tumours. The relative expression of these RNAs in tumour samples compared to corresponding normal tissue samples was calculated by dividing the mean expression level of all tumour samples by the mean value of the normal nonmalignant samples.

**Figure 3:**
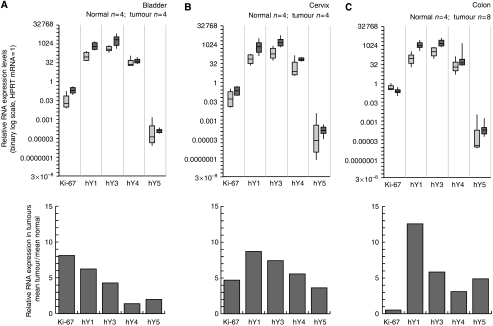

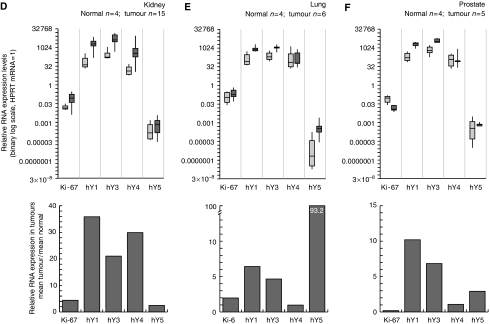
Tissue-specific overexpression of hY RNAs in human carcinomas and adenocarcinomas. Distributions of relative RNA expression levels and elevation of tumour-specific relative hY RNA expression are shown for the following tissues: (**A**) urinary bladder; (**B**) cervix; (**C**) colon; (**D**) kidney; (**E**) lung; (**F**) prostate. Data are presented as detailed for Figure 2.

To assess a role for hY RNAs in cancer, we determined the relative RNA expression levels in human solid tumours (42 samples) and corresponding healthy nonmalignant tissues (24 samples). These samples contain representatives from six different tissue types: urinary bladder, cervix, colon, kidney, lung and prostate. We first investigated the overall distributions of relative expression levels of Ki-67 mRNA and the four hY RNAs in all tumour and healthy tissue samples (Figure 2), and secondly differentiated these results according to the six different tissue types (Figure 3 and Table 1).

The overall range of relative Ki-67 mRNA expression overlaps between normal and tumour samples (Figure 2A), but the mean Ki-67 mRNA expression was 1.6-fold higher in the tumour samples compared with normal tissue samples (Figure 2B). This slight overall increase was not significant (*t*-test, two-tailed, unpaired, *P*=0.172). However, Ki-67 mRNA expression was strongly increased in tumours of the bladder, cervix, kidney and lung, whereas it was actually reduced in colon and prostate tumours (Figure 3).

The overall ranges of relative hY RNA expression show a partial overlap, with a general upward shift in expression level from normal to tumour samples (Figure 2A). Overall, the mean relative expression levels of all four hY RNAs are increased in tumours compared to normal tissue, ranging from 4-fold for hY4 to 13-fold for hY1 (Figure 2B). This increase was significant for each of the four hY RNAs (*t*-test, two-tailed, unpaired, *P*<0.005). Importantly, all hY RNAs were overexpressed in tumours of all six-tissue types investigated (Figure 3). However, we note three exceptions from the overall trend: the highest levels of hY1, hY3 and hY4 overexpression were observed in kidney tumours, a very high level of hY5 overexpression in lung tumours, and no or borderline overexpression was observed for hY4 RNA in bladder, lung and prostate tumours (Figure 3). Taken together, these data strongly suggest that human carcinomas (and adenocarcinomas) have elevated hY RNAs expression levels compared to the corresponding healthy tissue.

To determine the statistical significance of these results, we performed an analysis of variance (two-way ANOVA) on the original qRT–PCR data (Table 1). For each RNA in turn, we fitted a model with the explanatory factors malignancy type (tumour *vs* normal), tissue type (bladder *vs* cervix, colon, kidney, lung and prostate) and the interaction between malignancy type and tissue type.

For Ki-67 mRNA, the most significant factor was interaction between malignancy type and tissue type, indicating that relative expression levels vary both with malignancy type and with tissue type and, moreover, that the difference in expression between cancer and nonmalignant type varies with tissue type (Table 1). We conclude that while levels of Ki-67 mRNA can be linked to cancer, this link varies considerably depending on the tissue involved. This increase in expression of Ki-67 mRNA in some tumours is consistent with its established role as a biomarker for individual proliferating cells, whose contribution to overall tumour mass would vary between different tissues (Scholzen and Gerdes, 2000; Brown and Gatter, 2002).

In the ANOVAs of hY RNAs, the consistently dominant factor is malignancy type (Table 1), which ranges from highly significant (hY4 and hY5) to extremely significant (hY1 and hY3). This indicates a highly consistent pattern of expression, in which the relative expression levels of all four hY RNAs are increased in tumours relative to normal nonmalignant tissue. In addition, hY3 and, to a lesser extent, hY4 RNA, both exhibit significant tissue type terms, indicating some tendency for expression levels to vary with tissue type (see also Figure 3). In contrast to the expression of Ki-67 mRNA, all four hY RNAs reveal either nonsignificant or borderline significant interaction terms (Table 1), indicating a simple additive pattern where, for example, higher expression in cancer and higher expression in tissue X combine to produce very high expression in cancerous tissue X (Figure 3).

Taken together, these data establish that the expression of hY RNAs is significantly elevated in human cancers of the bladder, cervix, colon, kidney, lung and prostate. In particular, the extremely significant elevation of hY1 and hY3 RNA levels in these carcinomas (and adenocarcinomas) in all tissue types investigated identifies them as new cancer biomarkers.

### Functional requirement of hY RNAs for cell proliferation

**Figure 4:**
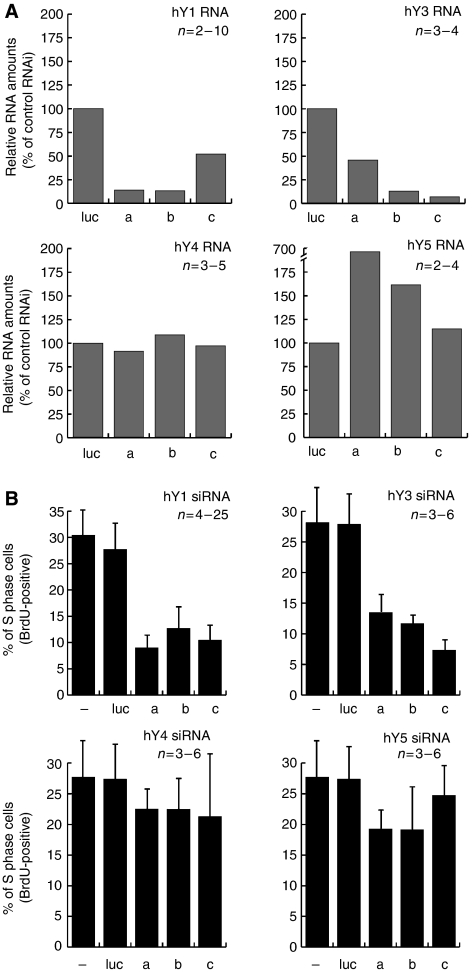
RNA interference against hY RNAs. (**A**) Quantification of RNA levels after RNAi. Three small interfering RNAs (designated as siRNAs a, b and c) directed against hY1, hY3, hY4 and hY5 RNA were transfected into proliferating HeLa cells. RNA was isolated at 48 h after transfection and the amounts of each targeted hY RNA relative to a calibrator RNA were determined by qRT–PCR. 5.8S rRNA was used as calibrator for hY1, and HPRT mRNA for the other hY RNAs. The expression of each target hY RNA after the experimental RNAi is shown as the percentage of the relative expression levels observed after a control RNAi against nontarget firefly luciferase mRNA. Mean values are shown for *n* independent experiments as indicated. (**B**) Quantification of replicating S phase cells after RNAi. At 47 h after transfection of asynchronously proliferating HeLa cells with the indicated siRNAs, replicating cells in the population were labelled for 1 h with BrdU. At 48 h, percentages of S phase cells incorporating BrdU into their chromosomal DNA were determined by immunofluorescence microscopy. Mean values and s.d. are shown for n independent experiments.

**Figure 5:**
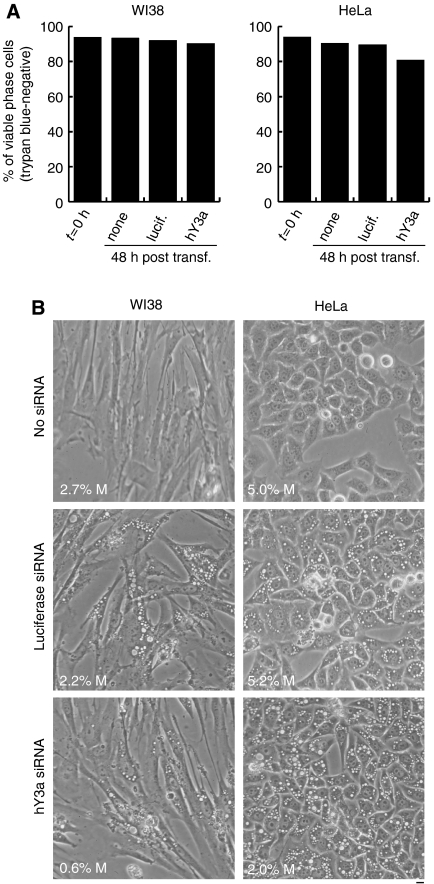
Depletion of hY3 RNA is cytostatic and does not cause cell death. (**A**) Cell viability assay. RNAi was performed on WI38 lung fibroblasts and HeLa cervical carcinoma cells. At 0 h and 48 h post transfection with the indicated siRNAs, percentages of viable cells were determined by measuring exclusion of the dye, trypan blue. (**B**) Cell morphology. Representative phase contrast micrographs of cells are shown at 48 h after transfection. The mitotic index (%M) for each of these cell populations was measured by counting >800 cells per sample and it is indicated at the bottom left of each field. Scale bar, 10 *μ*m.

In the next set of experiments, we investigated whether degradation of hY RNAs in proliferating human cells leads to an inhibition of cell proliferation. All four hY RNAs were expressed in several cell lines investigated (Supplementary Figure S1), in agreement with earlier reports (Hendrick *et al*, 1981; Pruijn *et al*, 1993). Our previous experiments have already established that RNA interference (RNAi) against hY1 RNAs is feasible in human cells, and we reported that degradation of hY1 RNA by two separate siRNAs results in a reduced proportion of replicating cells (Christov *et al*, 2006). We have therefore extended this analysis and conducted a systematic survey of RNAi on all four hY RNAs and analysed the consequences on cell proliferation (Figure 4) and cell viability (Figure 5).

For every hY RNA, we synthesised three distinct 21-nucleotide siRNAs (termed a, b and c) that target specific nucleotide sequences. Due to the small size of the hY RNAs and their partial nucleotide sequence conservation, these siRNAs therefore cover the entire sequences available for specific targeting. siRNAs were transfected separately into asynchronously proliferating HeLa cells, and the expression levels of each hY RNA were determined by quantitative RT–PCR after 48 h (Figure 4A). All three siRNAs specific for hY1 and hY3 RNA resulted in a 2–8 fold reduction in the amount of the targeted hY RNA (Figure 4A). In contrast, the relative expression levels of nontargeted 5S rRNA did not change more than about twofold in these experiments (data not shown). For hY4 and hY5 RNA we failed to achieve specific degradation with any of the siRNAs tested (Figure 4A).

Next, we analysed the effects of hY RNA degradation on the proportion of S phase cells in the transfected populations (Figure 4B). Transfection of any of the siRNAs directed against hY1 or hY3 RNA resulted in a two- and threefold reduction of S phase cells in the population. This reduction is statistically highly significant (*t*-test, two-tailed, unpaired, *P*<0.0001). In contrast, transfection of siRNA against control nontarget luciferase mRNA, or of inactive siRNAs directed against hY4 or hY5 RNA, did not result in a significant reduction in the proportion of S phase cells (Figure 5B; *t*-test, two-tailed, unpaired, *P*>0.02). To test for possible synergistic effects, we cotransfected cells with the two active siRNAs (against hY1 and hY3 RNA) together. This cotransfection did not reduce the proportion of S phase cells more than was achieved using either a single siRNA separately (Supplementary Figure S2). This observation can most likely be explained by the redundancy of hY RNAs, which can functionally substitute for each other in chromosomal DNA replication (Christov *et al*, 2006). Finally, we also transfected siRNAs directed against hY1 RNA into proliferating EJ30 bladder carcinoma, DU145 prostate carcinoma, ME180 cervical carcinoma and WI38 lung fibroblast cells. Degradation of hY1 RNA in these cells also showed a two- and threefold reduction of the proportion of S phase cells (Supplementary Figure S3). We conclude that the degradation of hY1 and hY3 RNAs causes significant inhibition of chromosomal DNA replication in cultured human cells.

This observation suggests that hY RNA degradation may result either in a cytostatic or in a cytotoxic inhibition of cell proliferation. To discriminate between these possibilities, we determined cell viability and morphology after RNAi (Figure 5). Mock transfections, or control transfections with a nontarget siRNA, did not have any effect on cell viability in any cell lines tested (Figure 5A). Importantly, degradation of hY3 RNA did not reduce the percentages of viable cells (Figure 5A). Identical results were obtained when hY1 RNA was degraded instead of hY3 RNA (data not shown). Furthermore, an assessment of cell morphology of the transfected cells provided no indication of cell death in any case (Figure 5B). However, we observed unspecific vacuolarisation of a subpopulation of transfected cells, irrespective of which siRNA was used, which can thus be ascribed to the experimental procedure (Figure 5B). In addition, the mitotic index was reduced between two- and threefold after degradation of hY3 RNA at 48 h after transfection, compared to the mock- and luciferase siRNA-transfected cells (Figure 5B). During the siRNA treatment against hY3 RNA, WI38 and HeLa cells only grew to cell densities of 50 and 59% of the mock-transfected controls, respectively. Taken together, these data demonstrate that degradation of hY RNAs in asynchronously proliferating human cells leads to a cytostatic, and not a cytotoxic, inhibition of cell proliferation.

## DISCUSSION

In this study, we have investigated the suitability of noncoding hY RNAs as new cancer biomarkers and potential targets for anti-proliferative intervention. We have analysed the relative expression levels of hY RNAs in human tumours and corresponding nonmalignant tissues by quantitative RT–PCR. We found that the relative expression levels of all four hY RNAs are significantly higher in carcinomas and adenocarcinomas of the urinary bladder, cervix, colon, kidney, lung and prostate than in corresponding nonmalignant tissues. Subsequently, we have performed systematic RNA interference against all hY RNAs and analysed the physiological consequence of their degradation in proliferating cells. We found that degradation of hY1 and hY3 RNAs in all human cell lines tested resulted in a significant and cytostatic inhibition of cell proliferation. Therefore, the correlative expression data in human tumours and corresponding tissues are complemented by functional studies, establishing that noncoding hY RNAs have potential both as new cancer biomarkers and as targets for anti-proliferative intervention.

The expression of functional wildtype hY RNA genes is very efficient in human cultured cells, with copy numbers of each expressed hY RNA reaching the order of 10^5^ molecules per cell (Hendrick *et al*, 1981; Christov *et al*, 2006). We determined here the relative hY RNA expression levels by qRT–PCR, using preparations of total soluble RNA from tissue samples and from cultured cells. The apparent relative expression levels of hY5 RNA in human tissues were several orders of magnitude below those seen in cultured human cells (Supplementary Figure S1; Christov *et al*, 2006). This difference is due in part to the use of differently primed cDNA libraries for the qRT–PCR amplification. cDNAs were prepared from tissue RNA by random priming and from RNA of human cultured cells by specific priming for logistical reasons. A side-by-side comparison of qRT-PCR amplifications using specifically and randomly primed cDNA libraries from HeLa cell extract resulted in about 100-fold reduced levels only of hY5 RNA in the randomly primed cDNA, but not of the other three hY RNAs (Supplementary Figure S4). This reduction was significant (*t*-test, two-tailed, unpaired, *P*=0.008), and may be explained by inefficient random priming of this shortest and mostly double-stranded hY RNA for cDNA synthesis. It remains a possibility that hY5 RNA may also be expressed at lower levels in human tissues compared to cultured cells.

This qRT–PCR method determines quantitatively the relative RNA expression levels averaged over all cells present in the entire tissue sample, or the entire population of cultured cells. Therefore, this method has the disadvantage of not being able to detect variations of hY RNA expression levels between individual cells within a given tissue sample. However, it is both rapid and quantitative, opening up exciting possibilities for high throughput screening applications and immediate computational analysis. Furthermore, the sensitivity of this approach would allow its application to the analysis of small volume biopsies in clinical situations.

The purified RNA samples from normal and malignant tissues used in this study were provided from commercial sources with pathological specifications of tissue type and diagnosis of malignancy. This information allowed us to find a highly significant elevation of hY RNA expression levels in tumour samples (i.e., carcinoma or adenocarcinoma), relative to normal tissue samples. Consequently, the relative hY RNA expression levels appear highly promising as sensitive novel cancer biomarkers, in particular hY1 and hY3 RNA. Considering the moderate number of cases (*n*=24 normal and *n*=42 tumour) and the selection of six tissues of epithelial lineage investigated, the work reported here constitutes a pilot study demonstrating the feasibility of using hY RNAs as novel cancer biomarkers. At present, we do not have access to patient history data, therefore we yet cannot evaluate the potential of relative hY RNA expression levels to be used to form clinical prognoses. A detailed clinical follow-up study with a large number of cases is now warranted in which individual case history would be linked to individual hY RNA levels in malignant and corresponding normal tissue samples.

Noncoding hY RNAs are functionally required for semiconservative DNA replication *in vitro* using template nuclei from mammalian late G1 phase cells (Christov *et al*, 2006; Krude, 2006). Degradation of either hY1 or hY3 RNA (or both) is feasible in human cells by transfection of specific siRNAs, leading to a significant reduction of the proportion of replicating cells in the treated population (S phase index), to a reduction of the mitotic index and to a cytostatic inhibition of cell proliferation. These data therefore support a functional requirement of hY RNAs for cell proliferation, extending the correlation between elevated hY RNA expression levels and the pathological diagnosis of a given tissue as cancer-derived. However, we were not able to degrade either hY4 or hY5 RNA by this approach, and thus unable to investigate their physiological role *in vivo*. The reason for this failure is unknown, but it may be linked to the predominantly double-stranded nature of hY4 and hY5 RNAs, which could interfere with efficient hybridisation of the siRNAs to its target sequences during RNAi (Rana, 2007). Likewise, we have been unable to degrade hY5 RNA *in vitro* by an antisense oligonucleotide-directed approach (Christov *et al*, 2006).

Our observation of a functional role for hY RNAs in mammalian chromosomal DNA replication and proliferation of human cultured cells is difficult to reconcile with knockout experiments in mice. Deletion of the Y RNA-interacting protein Ro60 leads to reduced levels of homologous mouse Y1 and Y3 RNA expression levels in adult brain tissue and embryonic stem (ES) cells (Chen *et al*, 2003; Xue *et al*, 2003). Ro60 knockout mice are viable and no proliferation defects were reported for the mutant ES cells. The different observations in these two mammalian systems may be due to different quantitative requirements of human and mouse Y RNAs for chromosomal DNA replication and cell proliferation, to physiological differences between model systems (e.g., mouse ES cells *vs* human somatic cells) or to different consequences of the pathway by which Y RNAs are degraded in these experiments (decreased RNA stability upon Ro60 depletion *vs* nucleolytic degradation by RNAi). In any case, future experimentation will be required to resolve these apparently paradoxical observations.

The feasibility of specifically degrading hY1 and hY3 RNA in intact human cells, thereby causing a cytostatic arrest of cell proliferation, suggests strongly that these RNAs should be considered as targets for therapeutic intervention. Future studies would now be required to assess the feasibility of this approach in animal models, for instance in mice. We have already shown that human Y RNAs can substitute for endogenous mouse Y RNAs in supporting chromosomal DNA replication in isolated mouse cell nuclei (Christov *et al*, 2006; Krude, 2006), indicating some degree of functional conservation of Y RNAs between primates and rodents. The next step would be to assess feasibility of RNAi-directed, antisense oligonucleotide-directed, or antagomir-directed degradation of Y RNAs in accessible tissues and their tumours in mice.

In conclusion, noncoding RNAs are expressed at significantly higher levels in several human solid tumours of epithelial origin (carcinomas and adenocarcinomas) compared to corresponding nonmalignant tissues. This property identifies them as novel cancer biomarkers with promising diagnostic potential. In addition, their functional requirement for cell proliferation in cultured cells identifies them as novel targets for the development of potential anti-proliferative intervention and cancer treatments.

## Figures and Tables

**Table 1:** Analysis of variance of relative RNA expression levels in human tissue samples between different diagnostic groups

	***P***				
**Type**	**Ki67 mRNA**	**hY1 RNA**	**hY3 RNA**	**hY4 RNA**	**hY5 RNA**
Malignancy	0.06	**9.7 × 10^−26^**	**1.7 × 10^−22^**	**6.3 × 10^−5^**	**1.9 × 10^−4^**
Tissue	**2.7** × **10^−7^**	0.19	0.002	0.03	0.09
Malignancy × tissue interaction	**1.9** × **10^−4^**	0.32	0.31	0.04	0.20

Relative expression levels for each RNA were obtained as raw ΔC*_t_* values from qRT–PCR analysis of 42 tumour and 24 normal tissue extracts. Individual data were grouped according to malignancy type (tumour *vs* normal), tissue type (bladder *vs* cervix, colon, kidney, lung and prostate) and interaction. ANOVA (two-way between groups) was performed separately for each RNA. *P*-values (test=*χ*^2^; model=Gaussian, glm) are shown for each type and their interaction. Highly significant results (*P*<0.001) are highlighted in bold.
