# Supplementary figures and images for: Noncoding human Y RNAs are overexpressed in tumours and required for cell proliferation
Source: Br J Cancer. 2008 Feb 19;98(5):981–8. doi: 10.1038/sj.bjc.6604254 (PMC2266855; doi:10.1038/sj.bjc.6604254)

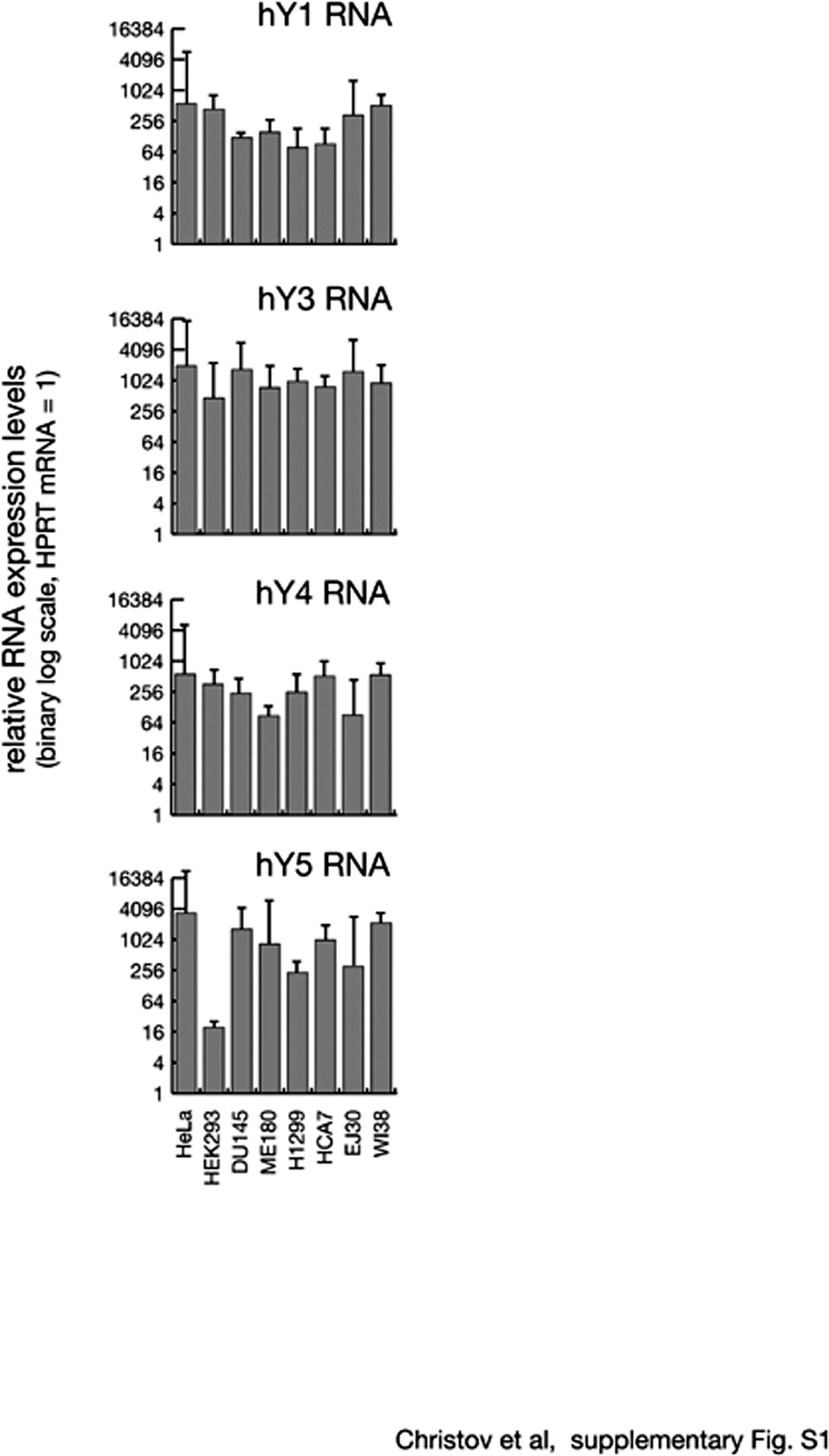

Supplement: Supplementary Figure S1 [file 6604254x1.tif]

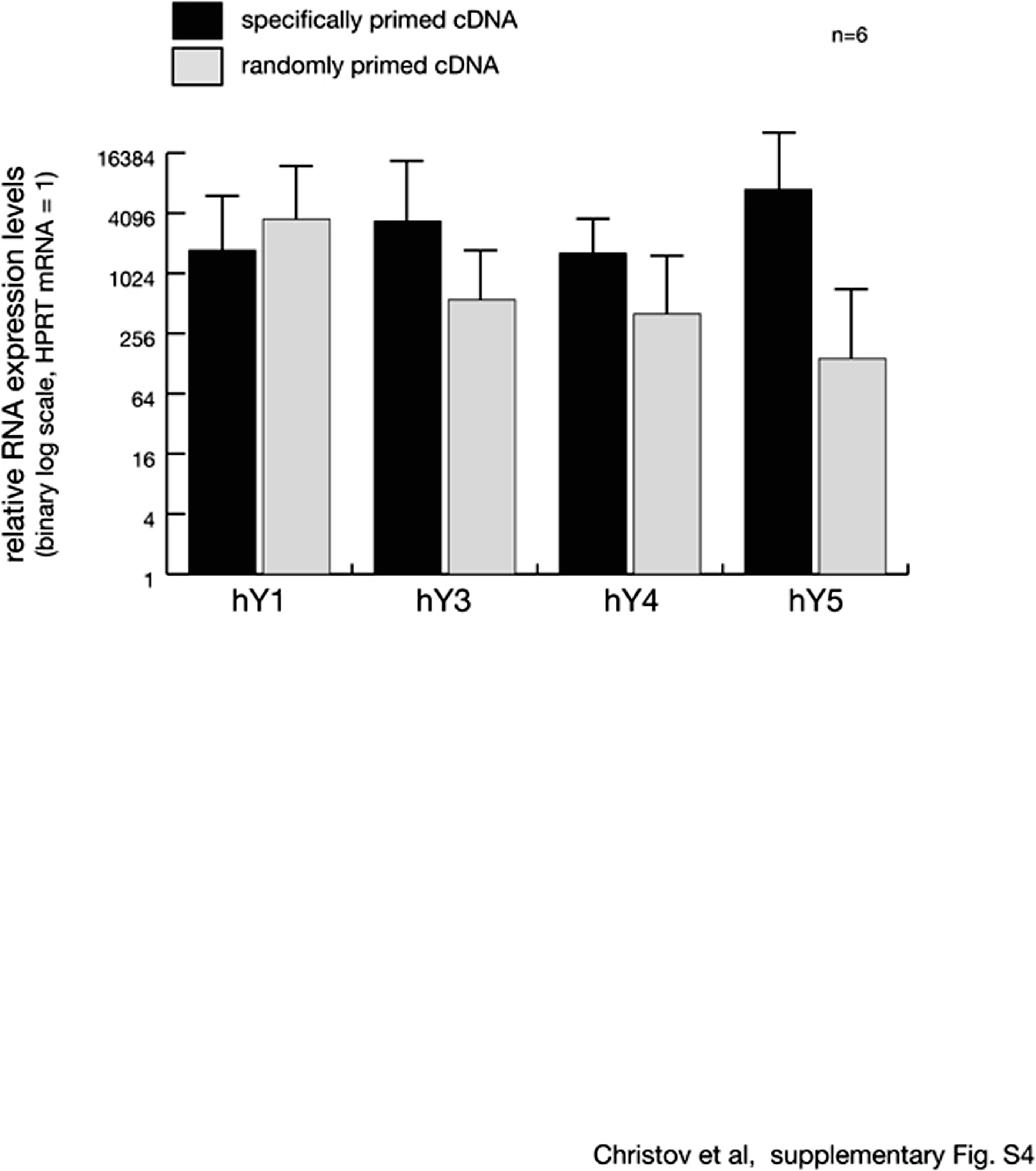

Supplement: Supplementary Figure S4 [file 6604254x4.tif]
