# Supplementary material for: Noncoding human Y RNAs are overexpressed in tumours and required for cell proliferation
Source: Br J Cancer. 2008 Feb 19;98(5):981–8. doi: 10.1038/sj.bjc.6604254 (PMC2266855; doi:10.1038/sj.bjc.6604254)
Supplement: Supplementary Material [file 6604254x5.doc]

# Supplementary material

**Non-coding human Y RNAs are over-expressed in tumours and required for cell proliferation**

Christo P. Christov, Elisabeth Trivier and Torsten Krude

# Legend to Supplementary Figure S1

Relative expression levels of hY RNAs in cultured human cell lines. Expression levels of the indicated hY RNAs were determined by qRT-PCR on a specifically primed cDNA in the 8 following human cell lines: HeLa cervical carcinoma, HEK293 adenovirus-transformed human embryo kidney, DU145 prostate carcinoma, ME180 cervical carcinoma, H1299 lung carcinoma, HCA7 colon carcinoma, EJ30 bladder carcinoma cells and WI38 lung fibroblasts. The expression level of HPRT mRNA is set as 1. All cell lines were asynchronously proliferating at the time of analysis. Mean values and standard deviations of 2-4 separate data acquisitions of n=2-6 independent experiments are shown.

# Legend to Supplementary Figure S2

RNA interference against hY1 and hY3 RNA is not additive. Quantification of replicating S phase cells after RNAi. At 47h after transfection of asynchronously proliferating HeLa cells with the indicated siRNAs, replicating cells in the population were labelled for 1h with BrdU. At 48h, percentages of S phase cells incorporating BrdU into their chromosomal DNA were determined by immunofluorescence microscopy.

# Legend to Supplementary Figure S3

RNA interference against hY1 RNA in different human cultured cells. Quantification of replicating S phase cells after RNAi. At 47h after transfection of asynchronously proliferating EJ30 bladder carcinoma, ME180 cervical carcinoma and DU145 prostate carcinoma cells, and WI38 lung fibroblasts with the indicated siRNAs, replicating cells in the population were labelled for 1h with BrdU. At 48h, percentages of S phase cells incorporating BrdU into their chromosomal DNA were determined by immunofluorescence microscopy. Mean values and standard deviations of n independent experiments are shown as indicated.

# Legend to Supplementary Figure S4

Inefficient qRT-PCR amplification of hY5 RNA from randomly primed cDNA. Randomly and specifically primed cDNA libraries were generated from the same preparation of total RNA from a cytosolic extract of proliferating HeLa cells. Relative expression levels of the indicated hY RNAs were determined by qRT-PCR. Mean values and standard deviations of n=6 independent experiments are shown.

**Supplementary Materials and Methods**

**Primer pairs used for RNA interference**

The following pairs of DNA oligonuceotides were synthesised (Sigma-Genosys) to direct generation of siRNAs *in vitro*:

*Firefly luciferase mRNA*

lucA: AACTTACGCTGAGTACTTCGACCTGTCTC,

lucB: AATCGAAGTACTCAGCGTAAGCCTGTCTC;

*hY1 RNA*

hY1a-A: TTATCTCAATTGATTGTTCACCCTGTCTC,

hY1a-B: GTGAACAATCAATTGAGATAACCTGTCTC;

hY1b-A: TGTTCTACTCTTTCCCCCCTTCCTGTCTC,

hY1b-B: AAGGGGGGAAAGAGTAGAACACCTGTCTC;

hY1c-A: TTACAGATCGAACTCCTTGTTCCTGTCTC,

hY1c-B: AACAAGGAGTTCGATCTGTAACCTGTCTC;

*hY3 RNA*

hY3a-A: TTTACAACTAATTGATCACAACCTGTCTC,

hy3a-B: TTGTGATCAATTAGTTGTAAACCTGTCTC;

hY3b-A: ATTTCTTTGTTCCTTCTCCACCCTGTCTC,

hY3b-B: AAGTGGAGAAGGAACAAAGAACCTGTCTC;

hY3c-A: AACTAATTGATCACAACCAGTCCTGTCTC,

hY3c-B: AAACTGGTTGTGATCAATTAGCCTGTCTC;

*hY4 RNA*

hY4a-A: TTATCAGAACTTATTAACATTCCTGTCTC,

hY4a-B: AATGTTAATAAGTTCTGATAACCTGTCTC;

hY4b-A: AAAGTTGGTATACAACCCCCCCCTGTCTC,

hY4b-B: AAGGGGGGTTGTATACCAACTCCTGTCTC;

hY4c-A: AACATTAGTGTCACTAAAGTTCCTGTCTC,

hY4c-B: AACTTTAGTGACACTAATGTTCCTGTCTC;

*hY5 RNA*

hY5a-A: TTATTGTTAAGUUGATTTAACCCTGTCTC,

hY5a-B: GTTAAATCAACTTAACAATAACCTGTCTC;

hY5b-A: AAGTTGATTTAACATTGTCTCCCTGTCTC,

hY5b-B: AAGAGACAATGTTAAATCAACCCTGTCTC;

hY5c-A: AACATTGTCTCCCCCCACAACCCTGTCTC,

hY5c-B: AAGTTGTGGGGGGAGACAATGCCTGTCTC
